# Development of primer-probe sets to rapidly distinguish single nucleotide polymorphisms in SARS-CoV-2 lineages

**DOI:** 10.3389/fcimb.2023.1283328

**Published:** 2023-12-07

**Authors:** Christopher S. Ealand, Bhavna G. Gordhan, Edith E. Machowski, Bavesh D. Kana

**Affiliations:** Department of Science and Innovation/National Research Foundation Centre of Excellence for Biomedical Tuberculosis (TB) Research, School of Pathology, Faculty of Health Sciences, University of the Witwatersrand and The National Health Laboratory Service, Johannesburg, South Africa

**Keywords:** SARS-CoV-2, single nucleotide polymorphisms, qPCR, minor groove-binding probes, COVID-19

## Abstract

Ongoing SARS-CoV-2 infections are driven by the emergence of various variants, with differential propensities to escape immune containment. Single nucleotide polymorphisms (SNPs) in the RNA genome result in altered protein structures and when these changes occur in the *S*-gene, encoding the spike protein, the ability of the virus to penetrate host cells to initiate an infection can be significantly altered. As a result, vaccine efficacy and prior immunity may be diminished, potentially leading to new waves of infection. Early detection of SARS-CoV-2 variants using a rapid and scalable approach will be paramount for continued monitoring of new infections. In this study, we developed minor groove-binding (MGB) probe-based qPCR assays targeted to specific SNPs in the *S*-gene, which are present in variants of concern (VOC), namely the E484K, N501Y, G446S and D405N mutations. A total of 95 archived SARS-CoV-2 positive clinical specimens collected in Johannesburg, South Africa between February 2021 and March 2022 were assessed using these qPCR assays. To independently confirm SNP detection, Sanger sequencing of the relevant region in the *S*-gene were performed. Where a PCR product could be generated and sequenced, qPCR assays were 100% concordant highlighting the robustness of the approach. These assays, and the approach described, offer the opportunity for easy detection and scaling of targeted detection of variant-defining SNPs in the clinical setting.

## Importance

Timely detection and identification of SARS-CoV-2 variants of concern is vital as emerging variants may influence diagnostic pickup, vaccine efficacy and disease transmission. The pace at which commercially available tests that distinguish SARS-CoV-2 variants is somewhat out-of-step with their rapid emergence. As a result, genomic surveillance using whole genome sequencing (WGS) is primarily the only way to identify new variants of concern (VOC), but this can be expensive and impractical to apply to every single clinical isolate. More cost-effective and simple tests to detect mutations in the SARS-CoV-2 genome are limited. Herein, we developed a robust set of PCR-based assays for SNP detection in the *S*-gene and report the methods to execute the assay, interpret the resulting data and validate findings. This approach alleviates the need for WGS by enabling scaling of targeted surveillance for VOC that are either currently circulating or archived from previous waves of infection.

## Introduction

SARS-CoV-2 is continually evolving, resulting in genomic mutation in the form of single nucleotide polymorphisms (SNPs) or deletions, predominantly in the spike (S) protein. These changes potentially confer increased infectivity or transmission, may evade the immune response either due to a reduced sensitivity to available vaccines or an inefficient memory response or be missed by routine diagnostic tests (Bal et al., 2021; Mcgrath et al., 2022; Ou et al., 2022; Liu et al., 2023). The World Health Organization (WHO) has designated five distinct variants of concern (VOC) together with numerous sub-variants that have been circulating for the last three years, which include Alpha, Beta, Gamma, Delta and Omicron. Variants being monitored (VBM) include Mu, Lamda, Kappa, Epsilon, Eta, Iota, Theta and Zeta (Flores-Vega et al., 2022; Aleem et al., 2023; Pandit and Matthews, 2023). Typically, viral variants have been classified according to unique mutations identified by whole genome sequencing (WGS) of residual SARS-CoV-2 positive clinical specimens and this approach remains the clinical gold standard (Tian et al., 2021; Volz et al., 2021a; Volz et al., 2021b; Kumar et al., 2022, Safari et al, Kim et al., 2020; Naqvi et al., 2020; Wang et al., 2020). However, WGS requires robust relationships between clinical diagnostic sites and laboratories with high throughput sequencing capacity and the expertise to analyse large-scale sequencing data sets in real-time (Grubaugh et al., 2021; Zare Ashrafi et al., 2023). In routine diagnostic settings, there is therefore an urgent need for inexpensive, targeted tools to assist with genomic surveillance of circulating variants (Anaclerio et al., 2021; Bezerra et al., 2021). Several RT-qPCR kits have already been developed for SARS-CoV-2 testing but varying and/or mutations in specific targets limit their use for routine surveillance (Garg et al., 2021; Kami et al., 2021; Vogels et al., 2021; Yaniv et al., 2021; Jain et al., 2023). The significant turnaround time to develop commercial tests significantly limits the ability of health systems to detect *en mass* and rapidly respond to VOC. Considering the essential role of the receptor binding domain (RBD) in the S protein, developing tools such as targeted qPCR assays combined with Sanger sequencing may offer an effective alternative to identify VOC (La Rosa et al., 2021; Li et al., 2021). For future VOC, it would be imperative to firstly identify all the defining mutations using WGS or to make predictions based on mutational hotspots in the genome to target unique SNPs for detection via qPCR assays. In this study, we designed and confirmed several targeted, probe-based qPCR assays using available WGS data to identify the following SNPs conferring mutations in the *S*-gene: E484K (present in Beta, Gamma, Eta, Iota and Mu), D405N (present in Omicron BA.2, BA.4, BA.5, BA.2.12.1, BA.2.75, BQ.1, XBB, XBB.1.5, XBB.1.16, CH.1.1, XBB.1.9, XBB.2.3 and EG.5.1), G446S (present in Omicron BA.1, BA.2.75, XBB, XBB.1.5, XBB.1.16, XBB.1.9, XBB.2.3, CH.1.1 and EG.5.1) and L981F (present in Omicron BA.1 only). To independently validate the performance of our qPCR assays, we used Sanger sequencing on a sub-set of clinical specimens to confirm the relevant SNPs in the *S*-gene. We have taken the approach of providing a historic narrative of probe development as variants emerged during the pandemic. Whilst particular probe and primer sets may not be relevant for currently circulating SARS-CoV-2 variants, we believe that the overall approach can easily be scaled for future emerging VOC to enable rapid genomic surveillance.

## Results

### Culturing and whole genome sequencing to confirm SARS-CoV-2 variants

**Figure 1:**
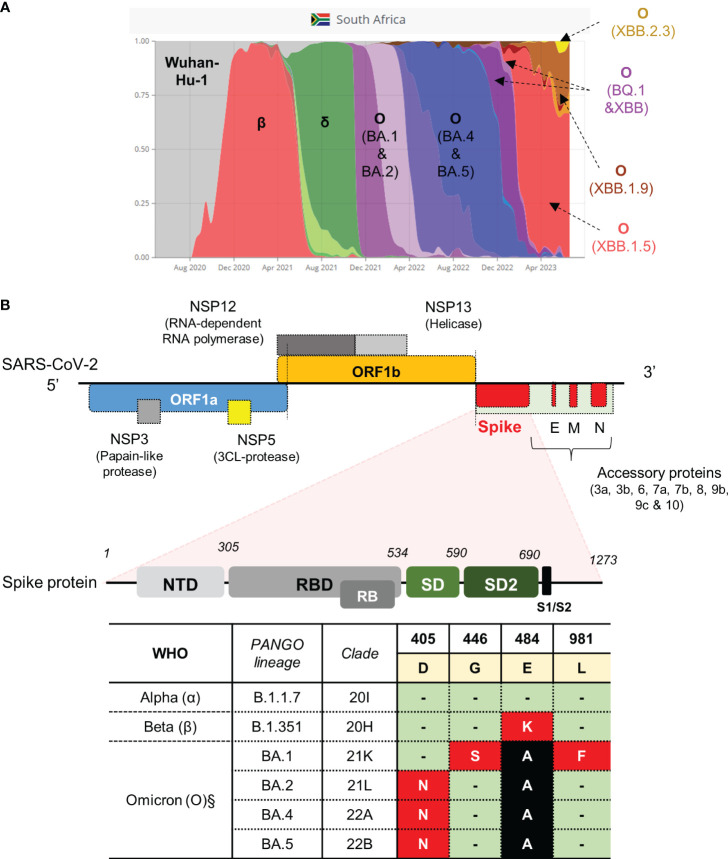
SARS-CoV-2 strains previously circulating in South Africa and schematic of the SARS-CoV-2 genome with qPCR assay targeting the *S*-gene. **(A)** Representative VOC between March 2020 and August 2023 (retrieved from https://www.covariantsorg). **(B)** Genomic architecture of SARS-CoV-2 virus. Regions in the gene encoding the spike protein highlighted to show domain structure (not drawn to scale). Regions in the *S*-gene with characterized SNPs at amino acid positions indicated. Amino acids occurring in the spike protein of the Wuhan-Hu-1 strain are highlighted in yellow. Three VOC classified according to PANGO lineage and clade (α, β and O sub-lineages) with corresponding mutations in the spike protein are shown in red and black blocks. Green blocks indicate no change. The table indicates the SNPs targeted for qPCR assay development (red blocks). The genome was adapted from (12, 40). Nucleic acid mutation profiles: D405N (GAT→AAT); G446S (GGT→AGT); E484K (GAA→AAA); L981F (CTT→TTT). ^§^Mutations are also present in newer sub-variants of Omicron, that emerged after BA.5, as highlighted in the main text.

We opted to develop tests for SARS-CoV-2 variants that were previously circulating in the South African context, each of which were associated with distinct waves of infection (**Figure 1A**). We previously obtained and cultured putative representative samples closest to Wuhan-Hu-1 (Clade 20A), Beta (Clade 20H) and Omicron (BA.1) (Clade 21K) strains which were confirmed using WGS as reported in our previous study (Gordhan et al., 2022). The strain that was most representative of Wuhan-Hu-1, hereon referred to as wild-type, contained a few amino acid substitutions in the ORF1a gene (A2710V), ORF1b (P314L; H604Y) and *S-*gene (V70F and D614G) but these were not sufficient to classify it as a different lineage. The genome of the Beta strain contained several amino acid substitutions in the *E*, *N*, *ORF1a*, *ORF1b*, *ORF3a* and *S* genes, with the defining mutations, E484K and N501Y, present in *S-*gene. Similarly, the genome of the Omicron (BA.1) strain contained several mutations in the *E*, *M*, *N*, *ORF1a*, *ORF1b* and *S* genes, with the G446S and L981F mutations present in the *S*-gene. These strains could therefore be used as positive controls in the development of our targeted qPCR assays in this study.

### Design strategy and optimization of qPCR assays for targeted surveillance

Several mutations resulting in amino acid substitutions in the *S*-gene were targeted for qPCR assay development (**Figure 1B**). We designed a multiplex qPCR assay to simultaneously distinguish between the nucleotides associated with the E484K amino acid substitution in one reaction. To simplify the approach, we subsequently designed a single-plex assay to only identify the SNPs conferring the D405N, G446S and L981F substitutions, respectively. Our approach utilized highly specific minor groove binding (MGB) probes, which can discriminate SNPs in the target of interest. The primers pair flanking each SNP of interest was tested for specificity using cDNA derived from the wild-type strain only. For E484K, combination of the *S*-F1 and *S*-R1 primer pair was specific and amplified a single amplicon following a melt curve analysis (**Supplementary Figure 1A**). Similarly, primers pairs flanking the D405N, G446S and L981F SNPs all amplified a single product (**Supplementary Figures 1C, D**). We next tested the specificity of qPCR assays containing fluorescently labelled MGB probes. In the case of E484K, the Cy5 labelled probe detected and amplified E484 (GAA codon) using cDNA derived from the wild-type and showed no cross-reactivity against cDNA derived from the Beta variant (known to harbor the E484K mutation) (**Supplementary Figure 2A**, *left panel*). Similarly, the ROX-labelled probe only detected and amplified 484K (AAA codon) using cDNA derived from Beta variant with no cross-reactivity to cDNA derived from the wild-type (**Supplementary Figure 2A**, *right panel*).

Given the potential for cross-reactivity and high costs associated with utilizing MGB-probes, we opted to use single-plex tests targeting SNPs in the *S*-gene for D405N, G446S and L981F. The qPCR assays were set up in a similar way, except that the MBG-probe targeting the wild-type codon was omitted from the reaction. At the time, we did not have access to purified RNA from Omicron (BA.2, BA.4 of BA.5) and as a result, there was no positive control for the D405N probe. However, as this mutation is absent in the Wuhan-Hu-1 and Omicron (BA.1) lineages, we interpreted the lack of amplification to likely indicate specificity for the D405N mutation. Indeed, when tested on wild-type or BA.1-derived cDNA, the Hex-labelled D405N probe failed to amplify above background levels. We surmised that this probe was specific for Omicron (BA.2/4/5) (**Supplementary Figure 2B**) which would be later confirmed using clinical specimens. The MGB-probes targeting G446S and L981F were highly specific and only amplified cDNA derived from cDNA viral strains known to contain the relevant mutations. More specifically, the FAM-labelled G446S probe amplified cDNA derived from Omicron (BA.1) with no cross-reactivity to wild-type cDNA (**Supplementary Figure 2C**). Similarly, the Cy5-labelled L981F probe showed high specificity for O (BA.1-derived) cDNA with no non-specific cross-reactivity against the wild-type cDNA (**Supplementary Figure 2D**). Overall, these multiplex and single-plex qPCR assays were able to accurately detect SNPs associated with various viral lineages that were purified from laboratory cultures. We therefore proceeded to test the performance and SNP calling utility on clinical samples.

### Assessing cross-reactivity of optimised probe sets against non-cognate sequences

Before testing performance of qPCR assays on residual clinical samples, we wanted to rule out any cross-reactivity between all four MGB-probe sets (i.e. E484K, D405N, G446S and L981F) and cDNA derived from either the wild-type, Beta or Omicron (BA.1) strains. The Cy5-labelled E484 probe remained specific for cDNA derived from the wild-type strain (C_T_ = 27.1) and did not amplify genomic material from the Beta or Omicron (BA.1) variants (C_T_ = 0) (**Supplementary Figure 2E**). Similarly, the Rox-labelled 484K probe only amplified cDNA derived from the Beta strain (C_T_ = 20.3) and failed to produce a signal when cDNA from the wild-type or Omicron (BA.1) strains were used (C_T_ = 0) (**Supplementary Figure 2E**). The Fam-labelled 446S and Cy5-labelled 981F MGB-probes only amplified cDNA derived from Omicron (BA.1) as expected (C_T_ = 22.9 and 24.6, respectively) (**Supplementary Figure 2E**). The Hex-labelled 405N MGB-probe failed to amplify cDNA derived from wild-type, Beta or Omicron (BA.1) (**Supplementary Figure 2E**) further supporting our conclusion that it would be specific for any VOC carrying the D405N mutation.

### Testing the multi-plex (E484K) and single-plex (G446S, L981F and D405N) qPCR assays on clinical samples

Residual swab material, collected during specific waves in South Africa, corresponded to Wuhan-Hu-1 (wild-type), Beta and Omicron lineages (**Figure 1A**). The ethics clearance numbers for these studies is provided in the methods section. Specifically, 95 de-identified, unique samples were collected and stored at 2 independent clinical sites between August 2020 and February 2022. Of these 95, 62 (65%) were collected during the second wave (dominated by Beta) and 33 (35%) were collected during the fourth wave (dominated by Omicron) (**Figure 1A**). Samples from the different waves were tested as independent batches using the SNP-specific qPCR assays.

**Figure 2:**
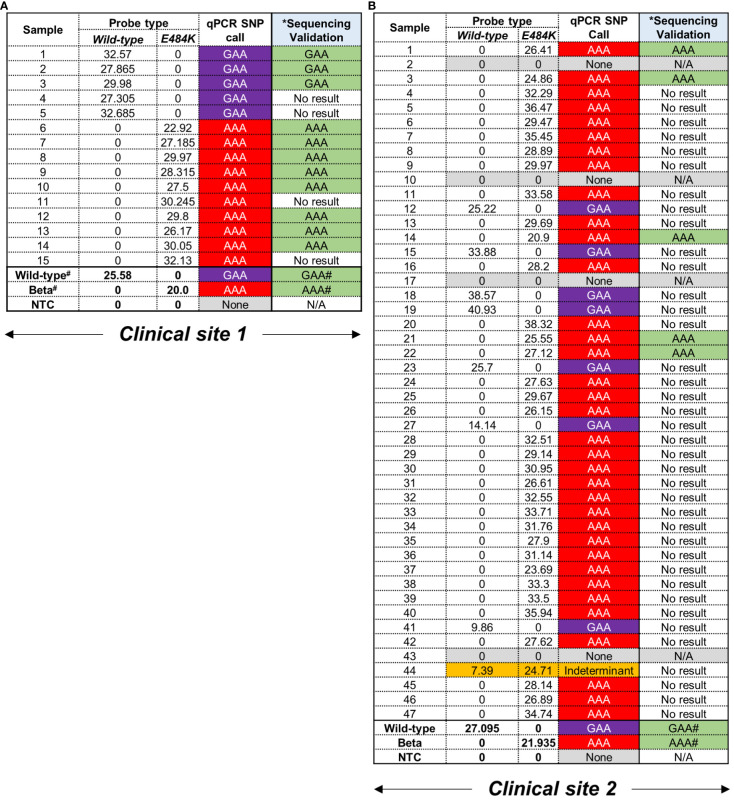
Performance of E484K probes using residual patient specimens. **(A)** Clinical site 1 retrieved 15 residual swab material for testing. Samples were designated as carrying either GAA (wild-type) or AAA (mutation) based on C_T_ values in the respective fluorescent channels, i.e. Wild-type = Cy5 and mutant = Rox. A C_T_ value < 38 was considered positive. #Positive controls for wild-type and Beta were previously confirmed using WGS. The codon at E484K (GAA→AAA) was independently confirmed using Sanger sequencing. ^*^The same RNA was used to create cDNA as template for a separate PCR and validation. No result was indicative an unusable chromatograph following sequencing. **(B)** Clinical site 2 retrieved 47 samples which were tested and assigned SNPs as described above.

Following screening with the E484K MGB-probes, 12/62 (19%) and 51/62 (82%) were deemed to harbour the GAA and AAA codons, respectively (**Figures 2A, B**). In this cohort, 4/62 samples (6%) could not be assigned to a strain-type likely due an insufficient amount of starting nucleic acid material in the original sample. Additionally, 1/62 (2%) could not be assigned to either the wild-type or mutant codon and was classified as ‘indeterminant’ (**Figure 2B**). In all cases, positive controls corresponding to genomic material derived from the wild-type or Beta variants were correctly identified by the E484K multiplex assay (**Figures 2A, B**). In order to confirm the mutations conferring the E484K amino acid substitution, the same RNA was used to prepare cDNA template to PCR amplify an 838 bp region in the *S*-gene (using the ‘*Spike_PCR_F* & *Spike_PCR_R*’ primer pair). This was designed to contain the potential mutation (**Supplementary Figure 3A**) for subsequent assessment using Sanger sequencing. One third of samples, 16/62 (26%) were successfully amplified (**Supplementary Figures 3B, C**). The lack of amplification in the remaining samples (74%) was likely due to low amounts of viral RNA isolated from residual specimens which were not affected in the more sensitive qPCR SNP calling application. Despite this low PCR yield, Sanger sequencing of the available amplicons demonstrated a 100% concordance with the SNP call using the E484K multi-plex qPCR assay (**Supplementary Figure 4**).

**Figure 3:**
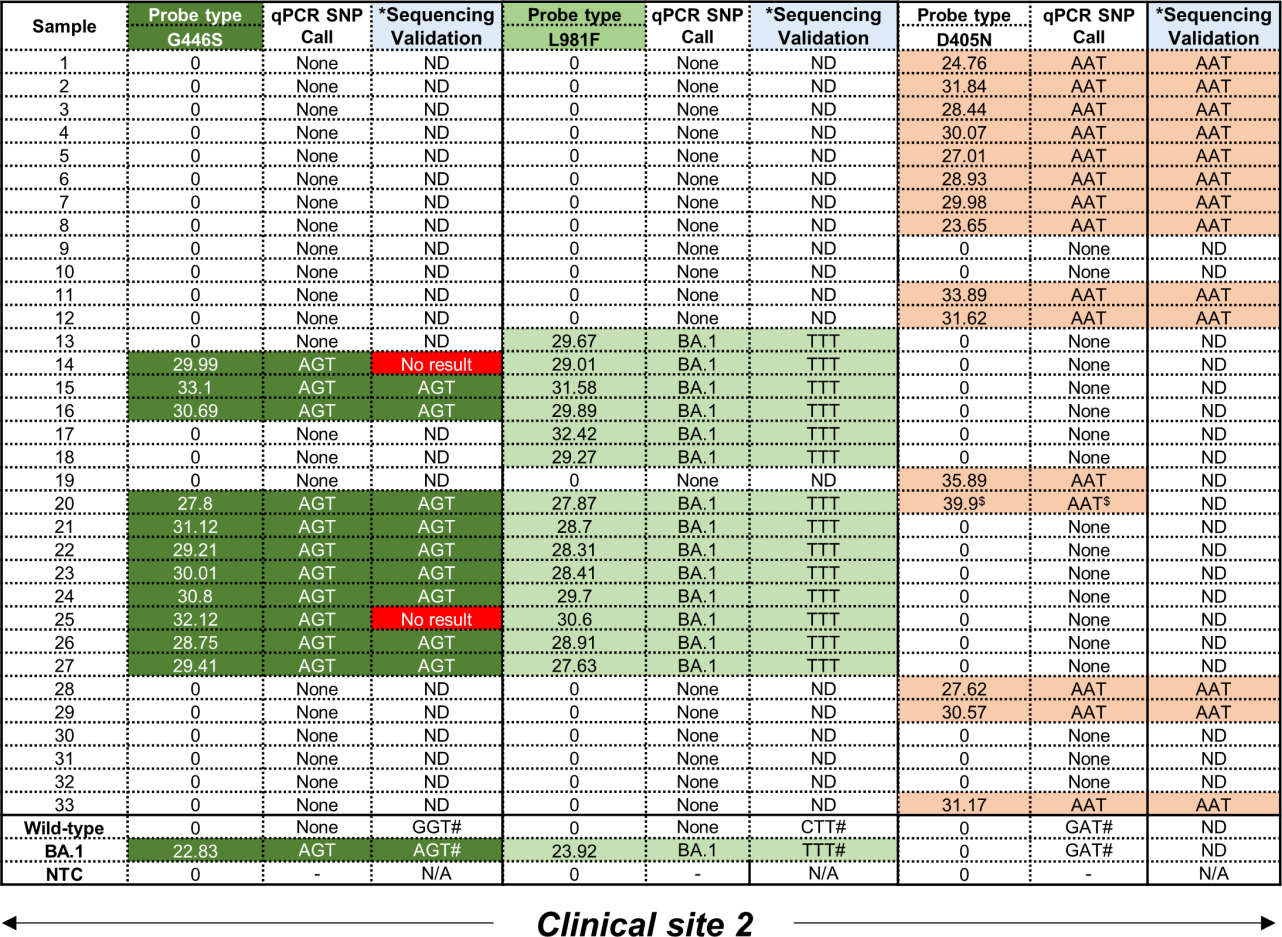
Performance of D405N, G446S and L981F single-plex qPCR assays using residual patient specimens. All samples were obtained from clinical site 2. For the G446S probe, samples shown in dark green were positive, based on C_T_ values in the FAM channel, for the AGT mutation. For the L981F probe, samples shown in light green were positive, based on C_T_ values in the Cy5 channel, for the TTT mutation. For the D405N probe, samples shown in orange were positive, based on C_T_ values in the Hex channel, for the AAT mutation. A C_T_ value < 38 was considered positive. Mutations were independently confirmed using Sanger sequencing to detect SNPs corresponding to G446S (GGT→AGT), L981F (CTT→TTT) and D405N (GAT→AAT). Where the sequencing failed, a ‘No result’ was reported. Where an amplicon was not obtained after PCR amplification, sequencing could not be done (ND). *The same RNA was used to create CDNA as template for a separate PCR and validation. ^#^Positive controls for the wild-type and Omicron (BA.1) strains were previously confirmed using WGS. ^$^Designated as a false positive as the CT value exceeded 38.

Following screening with the D405N, G446S and L981F single-plex qPCR assays to detect the GAT→AAT, GGT→AGT and CTT→TTT mutations, respectively, no wild-type codons were detected as expected as these were only designed to amplify the mutant codon (**Figure 3**). All 33 patient samples, collected exclusively at clinical site 2, were independently tested with all three qPCR assays followed by validation using Sanger sequencing. However, the validation approach had be altered as follows: because both flanking primer pairs, i.e. ‘*Spike_PCR_F & Spike_PCR_R*’ and ‘*Spike_PCR_F2 & Spike_PCR_R2*’ (**Supplementary Figure 3A**) failed to amplify the relevant regions in the *S*-gene, the smaller amplicons obtained using the qPCR flanking primer for each SNP (**Table 1**) were used instead. The specificity of these reactions using clinical specimens was confirmed by the general presence of single peaks following a melt curve analysis. Melt curves for the L981F flanking primers were less specific with what seemed like slightly larger, non-specific peak (**Supplementary Figures 5A–C**). Following assessment of the performance for the D405N probe, the AAT codon was amplified in 15/33 (45%) clinical specimens which was confirmed by Sanger sequencing in 13/15 translating into an 87% concordance. The remaining two samples could not be confirmed due to technical issues with the Sanger sequencing (**Supplementary Figure 6A**). Failure to make a SNP calling occurred in 18/33 (55%) which could be attributed to either too low a starting RNA concentration or an alternative codon in this position. For the G446S probe, the AGT codon was amplified in 11/33 (33%) clinical specimens but only 9 could be independently confirmed using Sanger sequencing (**Figure 3**; **Supplementary Figure 6B**). Failure to make a SNP call occurred in 22/33 (67%) samples for same reasons described above. For the L981F probe, the TTT codon was amplified in 14/33 (42%) clinical specimens, and all (100%) were confirmed by Sanger sequencing (**Figure 3**; **Supplementary Figure 6C**). Interestingly, in all the samples that had a SNP call of AGT (for the G446S-probe) and TTT (for L981F-probe), no AAT codons (for the D405N-probe) were detected, apart from one exception. Sample 20 was positive for all three mutations but the D405N result was deemed a false-positive as the C_T_ value was only detected in the final round amplification (C_T_ value > 38).

**Table 1:** Primers and probe sequences (with modifications) to identify SNPs associated with various SARS-CoV-2 lineages.

Assay/SNP target	Oligonucleotide	Sequence 5’ or 3’ modification indicated on probe (P)	T_m_ & amplicon size	Concentration
**E484K**	*S*-F1 *S*-R1 P:484_GAA P:484_AAA	5’-CAACTGAAATCTATCAGGCC-3’ 5’-TGCTGGTGCATGTAGAAGTT-3’ 5’-(Cy5)-GTAATGGTGTT **GAA** GGTT-(MGB)-3’ 5’-(Rox)-TGTAATGGTGTT **AAA** GGTTT-(MGB)-3’	59°C/161 bp	300 nM 300 nM 150 nM 150 nM
**D405N**	D405N_F^∞^ D405N_R P:405_BA.2	5’-AAGTGTTATGGAGTGTCTCCTACTA-3’ 5’-TTTGCCCTGGAGCGATTTGT-3’ 5’-(Hex)-CTGACTTCATTACCTCTAA-(MGB)-3’	62°C/112 bp	300 nM 300 nM 150 nM
**G446S**	G446S_F^∞^ G446S_R P:446_BA.1	5’-GGCTGCGTTATAGCTTGGAA-3’ 5’-ACCGGCCTGATAGATTTCAGTTG-3’ 5’-(Fam)-TCTTGATTCTAAGGTTAGTGGT-(MGB)-3’	64°C/138 bp	300 nM 300 nM 150 nM
**L981F**	L981F_F^∞^ L981F_R P:981_BA.1	5’-CGCTTGTTAAACAACTTAGCTCCAA-3’ 5’-TGCACTTCAGCCTCAACTTTG-3’ 5’-(Cy5)-TCAAGACGTGAAAAGATATCAT-(MGB)-3’	64°C/94 bp	300 nM 300 nM 150 nM

^∞^Also used for Sanger sequencing of qPCR amplicons for variant calling validation.

## Materials and methods

### Ethics statement

All methods were performed in accordance with the relevant guidelines and regulations for growing and handling of SARS-CoV-2 as approved by the Institutional Biosafety Committee of the University of the Witwatersrand (approval number: 20200502Lab). Where necessary, all experiments were conducted in a Biosafety Level 3 laboratory, registered with the South African Department of Agriculture Forestry and Fisheries (Registration Number: 39.2/NHLS-20/010). The use of human specimens, and residual swab material for SARS-CoV-2 surveillance, was authorized by the University of the Witwatersrand Ethics Committee (clearance numbers: M1911201, 200405 and 200313).

### Purification and whole genome sequencing of SARS-Cov-2 variants

Residual swab material from SARS-CoV-2 positive patients was obtained and cultured to obtain purified samples for each strain as outlined in our previous study (Gordhan et al., 2022). Briefly, a minimum of 200 µl was used to infect 1x10^6^ Vero E6 cells for 3-4 days at 37°C and 5% CO_2_. The cells were monitored daily for cytopathic effects (CPE) and when approximately 80% of the cells showed detachment from the bottom of the culture well, the supernatant was harvested. Total RNA was extracted from a minimum of 150 µl using the NucleoSpin Viral RNA kit (Macherey-Nagel) according to the manufacturer’s instructions. Total RNA was sent to Inqaba Biotechnical Industries (Pty) Ltd, a commercial NGS service provider for whole genome sequencing and strain assignment. Characteristic mutations associated with each variant were confirmed using https://covariants.org.

### Design of single- or multiplex RT-qPCR assays with minor groove-binding probes

Following the purification and strain assignment, various SNPs (and the corresponding amino acid substitutions) (Hodcroft, 2021) were targeted for detection using our in-house qPCR assays. All primer and probe sequences are listed in **Table 1**. More specifically, SNPs in the *S*-gene of SARS-CoV-2 associated with circulating VOC were selected for further analysis as follows: E484K (GAA→AAA); D405N (GAT→AAT); G446S (GGT→AGT) and L981F (CTT→TTT) (Aleem et al., 2023). The multiplex assays consisted of novel primer/probe sets in which the wild-type and mutant probes were labelled with two different fluorophores. In contrast, the single-plex assays consisted of novel primer/probe sets in which only the fluorescently-labelled mutant probes was used. For each assay, flanking primers in the region of the SNP of interest was tested for specificity using SYBR green chemistry (Agilent) and a melt curve analysis using the following cycling parameters: 98°C for 2min; 40x cycles (98°C for 5s, 59°C for 5s, 72°C for 5s plus plate read); 65-98°C with 0.5°C increment and plate read between each step. Single peaks in the melt curve analyses were indicative of adequate primer specificity.

Once flanking primers were optimized for amplicon specificity, MGB probes were included in the qPCR assays and tested on genomic material derived from purified viral samples (Gordhan et al., 2022). A two-step protocol was used to generate complimentary DNA (cDNA) for testing. Briefly, 5 µl of total RNA was mixed with 2 µl of a reverse primer mix (containing spike-specific primers at a final concentration of 2.5 µM) (**Supplementary Table 1**) made up to final volume of 13 μl with sterile water. Primers were annealed to RNA (94°C for 1.5 min, 65°C for 3 min, 57°C for 3 min) and then snap-cooled on ice. The primer-annealed RNA sample (12.5 µl) was then added to a 12.5 µl reaction containing Superscript IV (reverse transcriptase (RT), Invitrogen). After a brief centrifugation to pool all components, cDNA was synthesized by incubating reactions at 55°C for 15 min followed by a heat-inactivation at 80°C (as per the manufacturer’s instructions). All MGB probe-based qPCR assays were performed using Brilliant III Ultra-Fast QPCR Master Mixes (Agilent). One microliter of each cDNA sample was assessed in 20 µl volumes using an optimized thermal cycling profile (95°C for 3 min; 40x cycles (95°C for 15s, *x*°C for 60s plus a plate reading in the appropriate channels (Fam, Hex, Cy5 or Rox). Each reaction was performed in duplicate and no template control (ntc) was included. on All qPCR assays were performed on a Bio-Rad CFX96 real-time PCR machine (C1000 touch thermal cycler).

### Collection, processing and variant calling of samples derived from patient samples

This study used de-identified residual swab material (either nasopharyngeal or oropharyngeal sites) from patients who tested positive for SARS-CoV-2 by RT-qPCR in both public and private health medical diagnostic facilities in South Africa. Positive SARS-CoV-2 specimens with cycle threshold (C_T_) values <30 were selected such that total RNA yield for downstream testing could be maximized. A total of 95 samples were collected between March 2020 and February 2022. A minimum of 150 µl residual swab material was heat-inactivated at 70°C for 15 min and then used for total RNA extraction and cDNA synthesis (as described above). One microliter of cDNA was tested in each qPCR reaction, which was set up in duplicate.

### Sanger sequencing for independent confirmation of SNP calling

To confirm SNP mutations, specific regions of the *S*-gene were amplified (the same total RNA was used for cDNA synthesis) using FastStart *Taq* DNA polymerase (Roche) as per the manufacturer’s instructions. The following protocol was used: 95°C for 5min; 40x (95°C for 30s, 60-65°C for 30s, 72°C for 60-90s); 72°C for 7min; on a Bio-Rad T100 thermal cycler. Amplicons were separated on a 2% TAE agarose gel at 100V for 60-90min and gel-extracted using the NucleoSpin Gel and PCR Clean−up, Mini kit (Macherey-Nagel) as per the manufacturer’s instructions. In certain cases, where FastStart Taq DNA polymerase failed to amplify DNA for sequencing, smaller amplicons generated from the qPCR assays, in the absence of the MGB probe, were sequenced with the forward primers used in the qPCR reactions. The sequencing primers for E484K validation is listed in **Supplementary Table 1**. Sequencing chromatograms were analysed in SnapGene Viewer (Version 6.1.1) and sequences exported for alignment in SnapGene (Version 6.1.1) using the MUSCLE (MU ltiple S equence C omparison by L og- E xpectation) algorithm within SnapGene which is thought to achieve both better average accuracy and speed relative to ClustalW2 or T-Coffee (Madeira et al., 2022). Wuhan-Hu-1, Beta or Omicron (BA.1) sequences were used as a reference for the interrogation of genomes derived from clinical samples.

## Discussion

The emergence of novel SARS-CoV-2 variants continues to pose a significant risk to global health as these have the potential to confound diagnostic pickup, be more contagious and/or evade host immune protection provided by previous infections and vaccines. To date, the gold standard for genetic surveillance remains whole genome sequencing (WGS) but this is expensive, requires specialized laboratories and intensive bioinformatics analysis. In countries with limited resources for public health programs, this approach to SARS-CoV-2 surveillance is not feasible on a large scale. To circumvent this challenge, we, and others, have developed multi- and single-plex qPCR assays for targeted surveillance of variant-defining SNPs (Vogels et al., 2020; Erster et al., 2021; Hale et al., 2021; Vogels et al., 2021; Fabiani et al., 2022; Velu et al., 2022). The Thermo Fisher TaqPath assay was one of the first commercial tests to demonstrate the value of qPCR diagnostics for B.1.1.7 (Alpha) identification via the *S*-gene target failure (SGTF) (Brown et al., 2021). These assays offer immense clinical benefits as they are rapid and can be scaled for high-throughput analysis. One limitation of qPCR assays and probe design is that in order to identify lineage defining SNPs, the entire genome must first be sequenced. As a result, assay development will aways lag behind the emergence and identification of new mutations and VOC (Vogels et al., 2021). However, the development of commercial tests will be slower than deployment of in-house laboratory assays, as described herein, which can be easily scaled for use in diagnostic and surveillance laboratories. As SARS-CoV-2 is likely to be endemic in most geographical locations globally at this advanced stage of the pandemic, it is possible that VOC and sub-variants will share mutations making it difficult to use targeted qPCR assays for variant calling. However, as new VOC emerge, there is evidence that older lineages are completely replaced (Tegally et al., 2022; Lythgoe et al., 2023; Sah et al., 2023) thus supporting the development of targeted qPCR assays for surveillance. There is also evidence that the SARS-CoV-2 genome contains mutational hotspots, associated with higher rates of mutation (Goswami et al., 2021; Mullick et al., 2021; Omotoso et al., 2021; Ghosh et al., 2022). These areas should be focused on for the pre-emptive development of novel assays. A limitation to this approach could be that specific SNPs resulting in synonymous or non-synonymous mutations cannot be predicted with certainty. If several possibilities are incorporated into the qPCR design, the appropriate probes can be tested and implemented much faster. Whilst not without challenge, using this approach reduces the reliance on pre-existing WGS data.

In this study, we developed qPCR assays that can accurately detect known SNPs in the RBD of the *S*-gene in SARS-CoV-2, including E484K, D405N, G446S and L981F. Several other mutations in this region may serve as targets for novel assay development (Boehm et al., 2021; Khan et al., 2021; Wang et al., 2021; Yaniv et al., 2021; Cao et al., 2022; Pastorio et al., 2022; Scarpa et al., 2022). In other similar studies, qPCR probes were utilized to detect mutations in the SARS-CoV-2 genome. For example, a highly sensitive one-step reverse-transcription-quantitative PCR assay was developed for viral detection in wastewater which monitored the E484K mutation amongst others (Raya et al., 2023). Nasir and colleagues were able to track SARS-CoV-2 variants during pandemic waves, in a low-resource setting, using RT-PCR (Nasir et al., 2023). This study used commercially available RT-PCR assays to track specific mutations (i.e. N501Y, A570D, E484K, K417N, L452R, P681R and ΔH69/V70) to VOC genomic surveillance. Furthermore, the specific primer/probe sequences are not provided, merely the fluorescent channels and how to interpret the assay results are detailed. A different study designed two in-house variant screen RT-PCR multiplexed assays, with MGB probes, targeted against known mutations in the spike gene to rapidly distinguish emerging VOC (Moisan et al., 2023). The first assay targeted the ΔH69/V70 deletion and the N501Y mutation simultaneously while the second assay targeted the E484K, E484Q and L452R mutations simultaneously. Clinical samples were sequenced with targeted NGS and screened with these RT-PCR assays and showed a ~99.5% concordance. Another study designed two flexible, multiplexed RT-qPCR platforms for small- and large-scale screening, using MGB probes, to detect the L452R, E484K, N501Y and ΔH69/V70 deletion in clinical samples from Denmark (Spiess et al., 2023). Relative to our study, a similar approach was taken where the multiplexed assays comprised two probes – one to detect the wild-type nucleotide sequence and one to detect the mutation. To validate assay performance, variant calls were compared to WGS data. Our approach of using Sanger sequencing lends itself to better utility in resource limited settings. Other approaches for genomics surveillance, including the commercially available Seegene Allplex SARS-Cov_2 Variants I (E484K, N501Y and ΔH69/V70) and II (L452R, W152C, K417T, and K417N) assays, have opted to sequence the entire S-gene to identify VOC (Liotti et al., 2022). Clearly, whilst the approach to identifying SNPs using qPCR assays is not unique, the context in which these were developed must be considered. Under pandemic conditions, the availability of commercial kits in resource-limited settings was significantly affected, particularly when the E484K and N501Y mutations arose in the Alpha (α) and Beta (β) lineages. Moreover, the pipeline for novel assay development was slow and unless in-house qPCR assays were developed and tested in real-time, VOC could not be identified with the rapidity required to engage necessary public health interventions. These combined challenges, were particularly acute in Africa, necessitating that we developed our own qPCR assays for targeted surveillance. In this regard, we optimised the performance of each assay using genomic material from purified SARS-CoV-2 strains and then assessed in SNP calling in residual clinical specimens previously shown to be positive. In contrast to our approach which detects mutations through positive amplification, the failure to amplify certain regions may also serve as a proxy for novel variants. For example, the spike gene target failure (SGTF) genomic signature was shown to be accurate in identifying the Alpha and Omicron VOC (Brown et al., 2021; Mcmillen et al., 2022; Scobie et al., 2023). A limitation to using target dropouts for the detection of novel VOC is that it is a negative screen with no way to definitively rule out false negatives, apart from including a sample adequacy control (SAC) in the same test. Therefore, by using qPCR probes designed to detect specific SNPs, the possibility of obtaining false negatives is minimized.

In our qPCR assay, SNP calling of the E484K assay were performed using multi-plex qPCR assays whereas the remaining assays were only designed to detect the respective SNPs associated with amino acid substitutions. A limitation of the single-plex assays is that while it would be able to detect the SNP of interest, if two or more VOC share the mutation, it would not be possible to make a call on the viral lineage. Despite this limitation, we showed that all probes were specific for their cognate sequences and to independently confirm the SNP calling, regions harbouring respective mutation in the *S*-gene were amplified and Sanger sequenced. Other studies have used either Sanger sequencing or WGS for confirmation (Erster et al., 2021; Aralis et al., 2022; Moisan et al., 2023; Spiess et al., 2023; Zare Ashrafi et al., 2023). A large proportion of samples, particularly from Clinical Site 2, could not be confirmed as PCR failed to generate amplicons for sequencing. These specimens yielded a clear signal using the PCR diagnostic assay, which could be attributed to the enhanced sensitivity of qPCR assays relative to that used to generate amplicons for sequencing. The regions amplified for sequencing were significantly larger (838 – 1172 bp) relative to that used for the diagnostic PCR (ca. 94-138 bp) and amplification was performed with a standard DNA polymerase, which presumably had a lower sensitivity than the DNA polymerase used in the qPCR assay. We surmised that the starting amounts of cDNA were too low in these samples to be adequately amplified. Despite this, where sequence data was generated, there was excellent concordance between the SNP call and the sequencing result. Overall, the application of the qPCR assays described herein can be used to screen specimens containing these specific SNPs with relative ease. The approach is simple and inexpensive compared to WGS and other commercially available kits. Moreover, MGB-probes can be used on any open qPCR/diagnostic platform. The data analysis and interpretation of SNP assignments is easy, and confirmation can be done using amplicons generated in the qPCR reaction if required. Moreover, the reliance on commercially available tests, which take long to develop and do not necessarily address key healthcare needs in developing nations, is minimized making it particularly useful in resource-limited settings. In conclusion, our proposed method is practical and provides rapid monitoring of VOC-defining SNPs in archived clinical specimens and the methodology can be applied to future variants as the COVID-19 pandemic continues to evolve.

## Data availability statement

The original contributions presented in the study are included in the article/**Supplementary Materials**. Further inquiries can be directed to the corresponding author.

## Ethics statement

The studies involving humans were approved by Institutional Biosafety Committee of the University of the Witwatersrand (approval number: 20200502Lab) University of the Witwatersrand Ethics Committee (clearance numbers: M1911201, 200405 and 200313). The studies were conducted in accordance with the local legislation and institutional requirements. The participants provided their written informed consent to participate in this study.

## Author contributions

CE: Data curation, Formal Analysis, Investigation, Methodology, Writing – original draft, Writing – review & editing. BG: Data curation, Investigation, Methodology, Writing – review & editing. EM: Methodology, Writing – review & editing. BK: Conceptualization, Funding acquisition, Resources, Supervision, Writing – review & editing.
