# Supplementary material for: Development of primer-probe sets to rapidly distinguish single nucleotide polymorphisms in SARS-CoV-2 lineages
Source: Front Cell Infect Microbiol. 2023 Dec 7;13:1283328. doi: 10.3389/fcimb.2023.1283328 (PMC10733533; doi:10.3389/fcimb.2023.1283328)
Supplement: Supplementary file 1 [file DataSheet_1.pdf]

Supplementary Table 1. Primers for cDNA synthesis and Sanger sequencing for independent validation of SNP calling.

Oligonucleotide	Application	Sequence (5’-3’)	Amplicon size
Spike_PCR_F	Amplification of region in *S*-gene containing D405N, G446S, E484K and N501Y	TGTGTTGCTGATTATTCTGTCC	838 bp
Spike_PCR_R#		GAACCTGTAGAA TAA ACACGCC	
Spike_PCR_F2	Amplification of region in *S*-gene containing L981F	GGCGTGTTTATTCTACAGGTTC	1172 bp
Spike_PCR_R2#		GATTAGCAGAAGCTCTGATT	
Seq_F2	Sanger sequencing of E484K and N501Y SNPs	AGAGAGATATTTCAACTGAA	N/A
Seq_R2	Sanger sequencing of E484K and N501Y SNPs	GTGCATGTAGAAGTTCAAAA	N/A

#Also used for cDNA synthesis with Superscript IV.

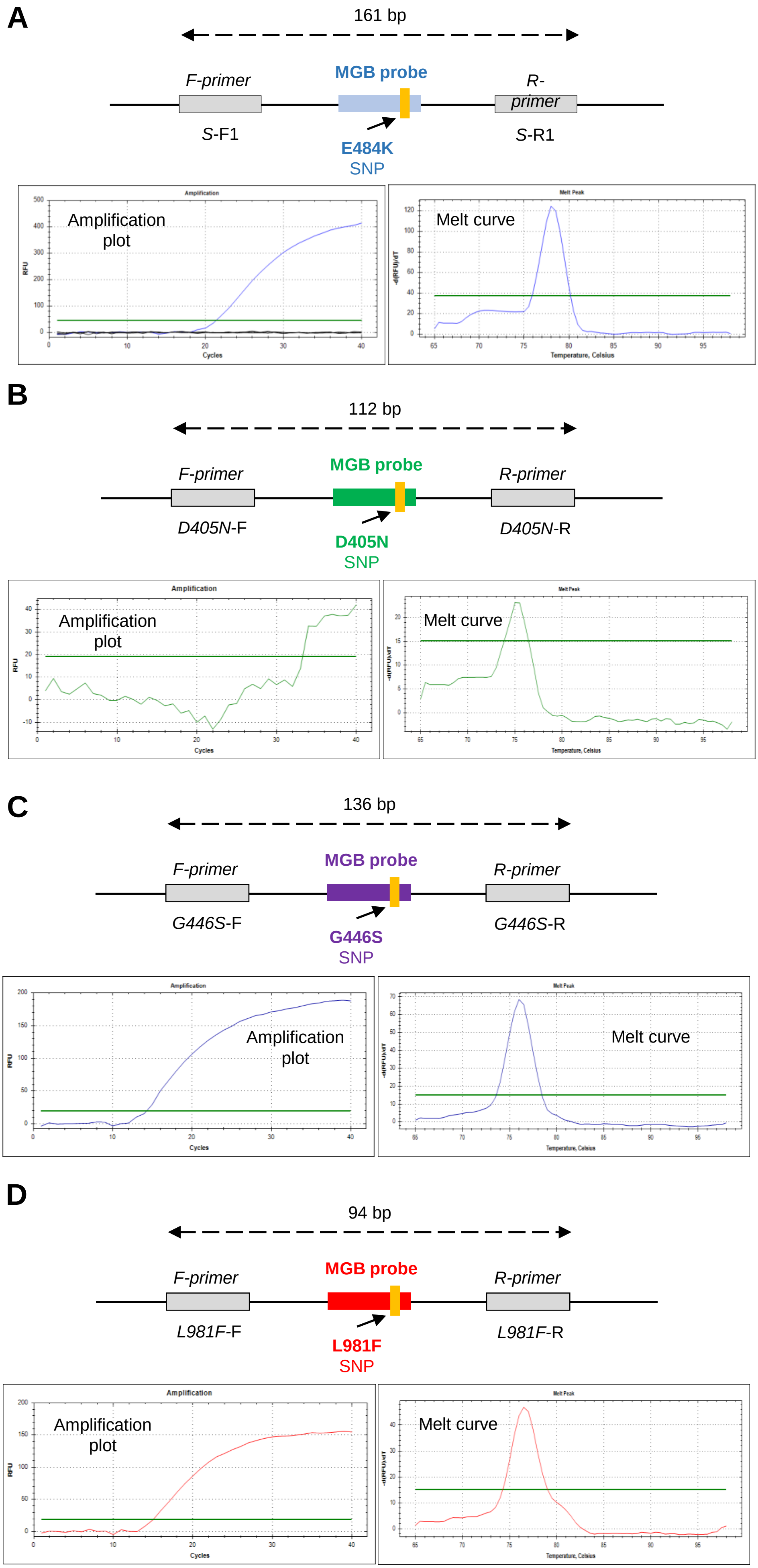

Supplementary Figure 1. Schematic presentation of primer pair and MGB probes targeting the E484K, D405N, G446S and L981F SNPs in the *S*-gene. (A) Grey boxes represent primer pair ***S*-F1R1**. Blue box represents the fluorescently labelled MGB probe targeting the E484K SNP (yellow line). Amplification plot and melt curve analysis (SYBR green chemistry) of *S*-F1R1 primer pair. (B) Primer-probe design, amplification plot and melt curve analysis for **D405N**. (C) Primer-probe design, amplification plot and melt curve analysis (SYBR green chemistry) of **G446S**. (D) Primer-probe design, amplification plot and melt curve analysis of **L981F**. In all cases, cDNA derived from the wild-type virus was used as template. Single peaks were observed in a melt curve analysis suggesting that primer pairs were highly specific. Representation of the genomic regions and positions of primers/probes targeted for assay development are not drawn to scale.

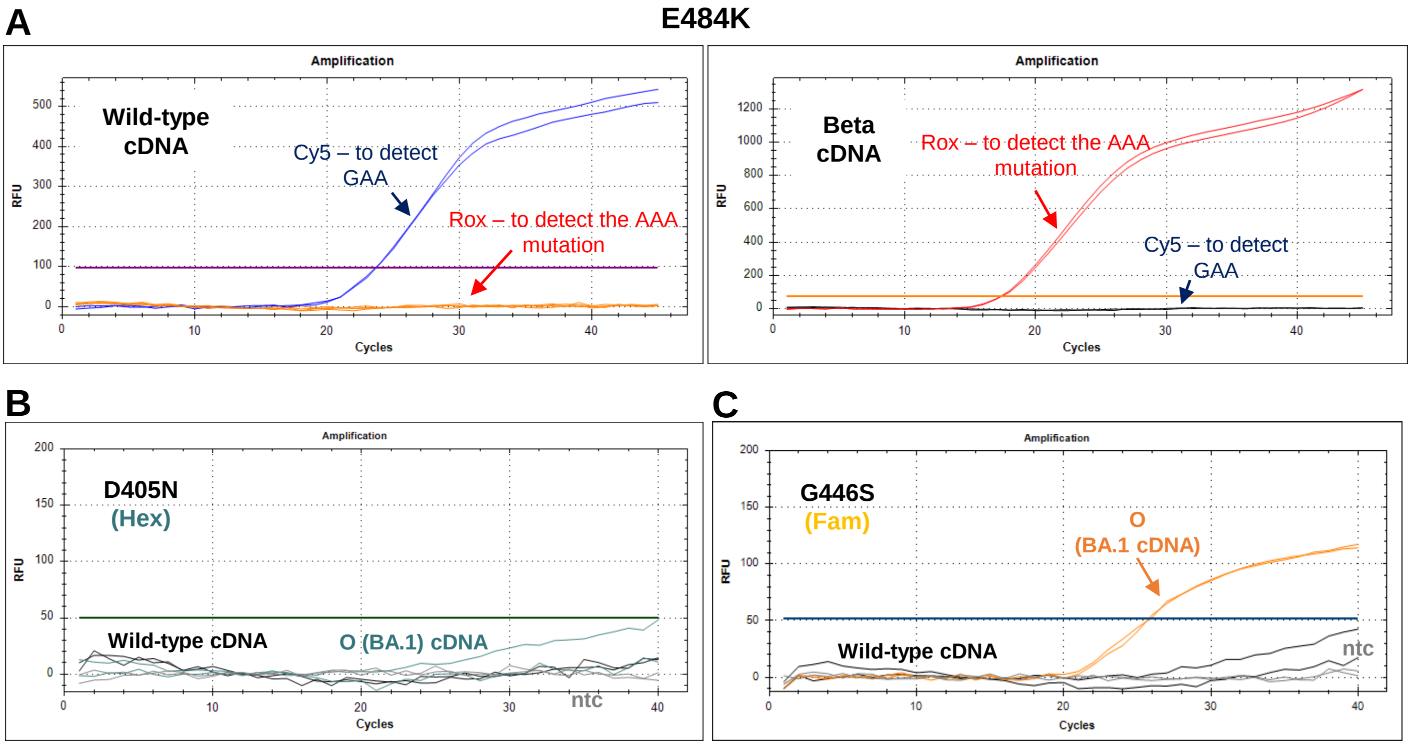

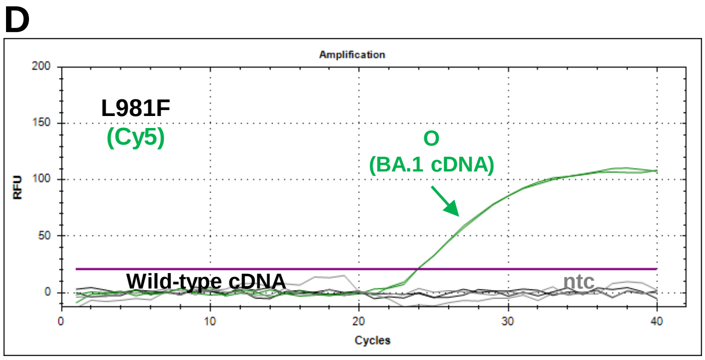

E

Strain	E484K		G446S	L981F	D405N
	Cy5	*Rox*	*Fam*	*Cy5*	*Hex*
Wild-type	27.1	0	0	0	0
Beta	0	20.3	0	0	0
Omicron (BA.1)	0	0	22.9	24.6	0

Supplementary Figure 2. Optimisation of E484K multiplex and D405N/G446S/L981F single-plex qPCR assays. (A) Amplification curves of fluorescence intensity versus cycle threshold (C_T) with E484K primer-probe sets. The GAA (Cy5) and AAA (Rox) MGB probes targeting the SNPs were combined in a single reaction and tested using cDNA derived from either of the two strains. Both MGB probes were highly specific and only amplified cDNA containing its cognate SNP. (B) Amplification curves of fluorescence intensity versus cycle threshold (C_T) with D405N primer-probe set. The D405N MGB probe (**Hex**) did not amplify cDNA from either template tested. (C) Amplification curves of fluorescence intensity versus cycle threshold (C_T) with G446S primer-probe set. The G446S MGB probe (**Fam**) strongly amplified cDNA containing its cognate SNP. (D) Amplification curves of fluorescence intensity versus cycle threshold (C_T) with L981F primer-probe set. The L981F MGB probe (**Hex**) strongly amplified cDNA containing its cognate SNP. The no template control (**ntc**) reactions were flat and void of any contaminating DNA. All qPCR reactions were set up in duplicate. (E) All MGB-probes were tested for cross-reactivity against non-cognate sequences. Cross-reactivity was assessed as amplification in the form of a C_T value. Fluorescent labels on each probe indicated below probe name.

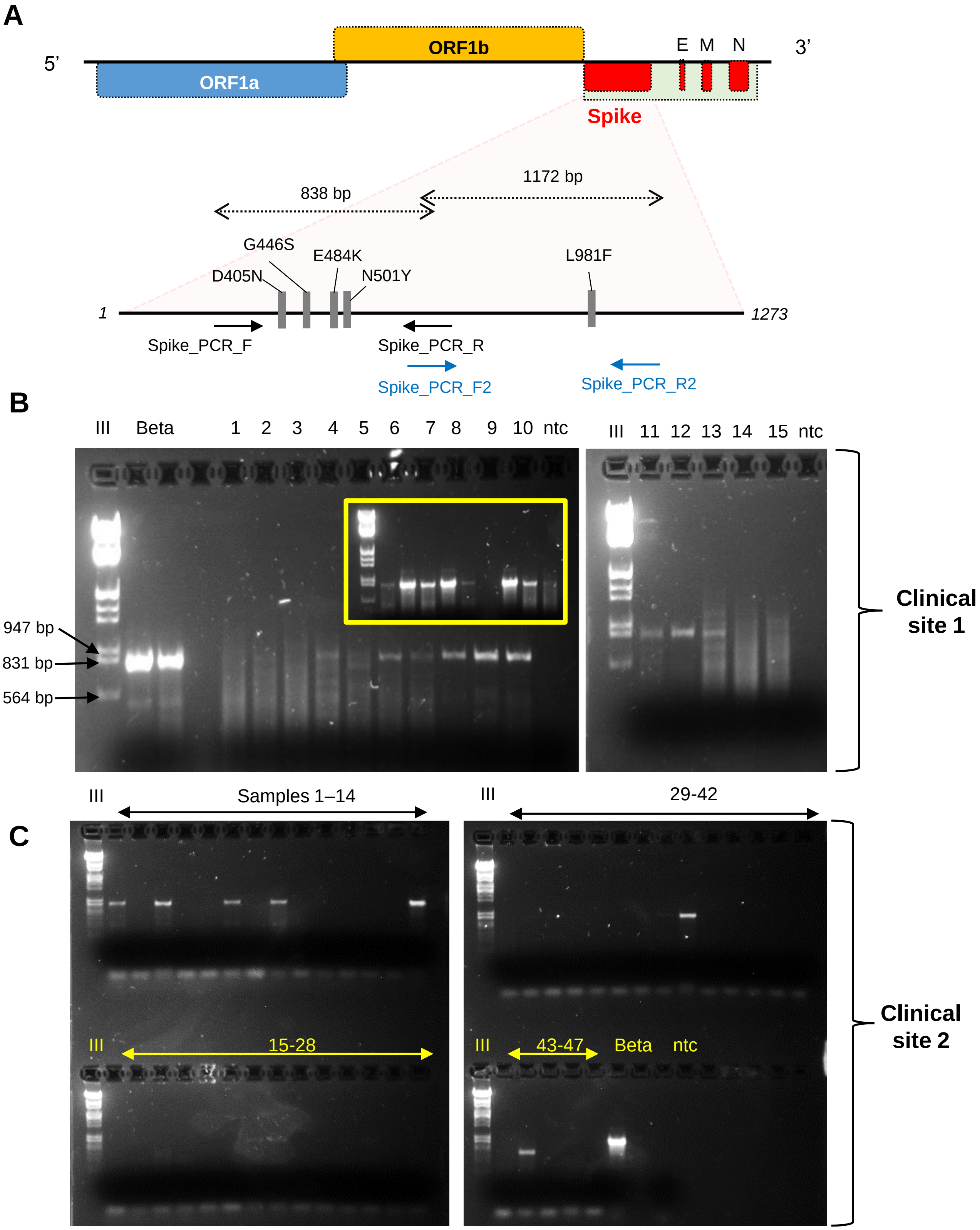

Supplementary Figure 3. Strategy to validate results of qPCR assay SNP calls using Sanger sequencing. (A) Primers were designed to flank the SNPs of interest (as indicated) to amplify either an 838 or 1172 bp region in the *S*-gene. (B) PCRs from the 15 residual swab samples at **clinical site 1**. PCRs for samples that yielded smears were repeated to increase specificity *(Inset, yellow border)*. (C) PCRs from the 47 residual swab samples at **clinical site 2**. For both batches, positive and negative controls included genomic material from the purified Beta variant and a no template control (ntc), respectively. PCR amplicons were separated on a 2% agarose gel and sized using DNA molecular weight marker III (Roche). The fragments of interest (838 bp) were gel-extracted and purified before Sanger sequencing.

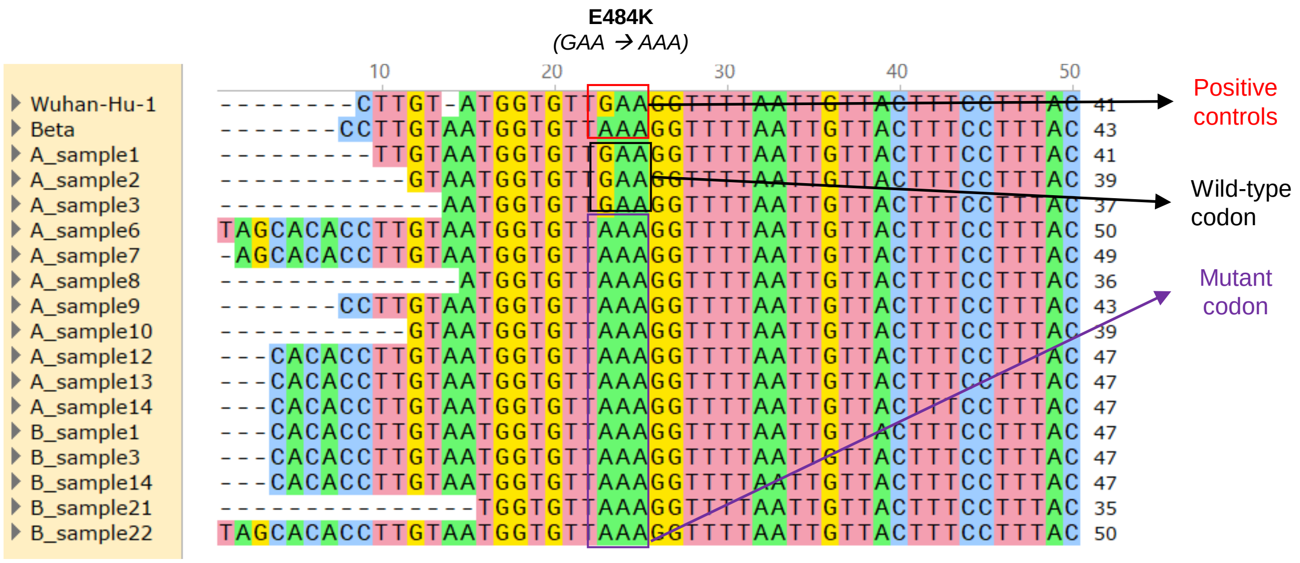

Supplementary Figure 4. Sequence alignment for SNP calling and assay validation for E484K probes using Sanger sequencing. Samples were aligned to sequences corresponding to the wild-type or Beta strains which served as positive controls (E484K SNP = GAA → AAA) [Red blocks]). Clinical sample names were annotated according to the clinical sites from which they were collected, i.e. A or B in the prefix corresponded to clinical site 1 or 2, respectively (see Figure 2). In all cases, where the E484K qPCR assay assigned a wild-type or mutant codon, the RNA nucleotide sequences showed 100% concordance. Samples with validated SNP calling are shown in the **black** or **purple** rectangles. Chromatographs and nucleotide alignments were performed using SnapGene (Version 6.1.1) using MUSCLE algorithm.

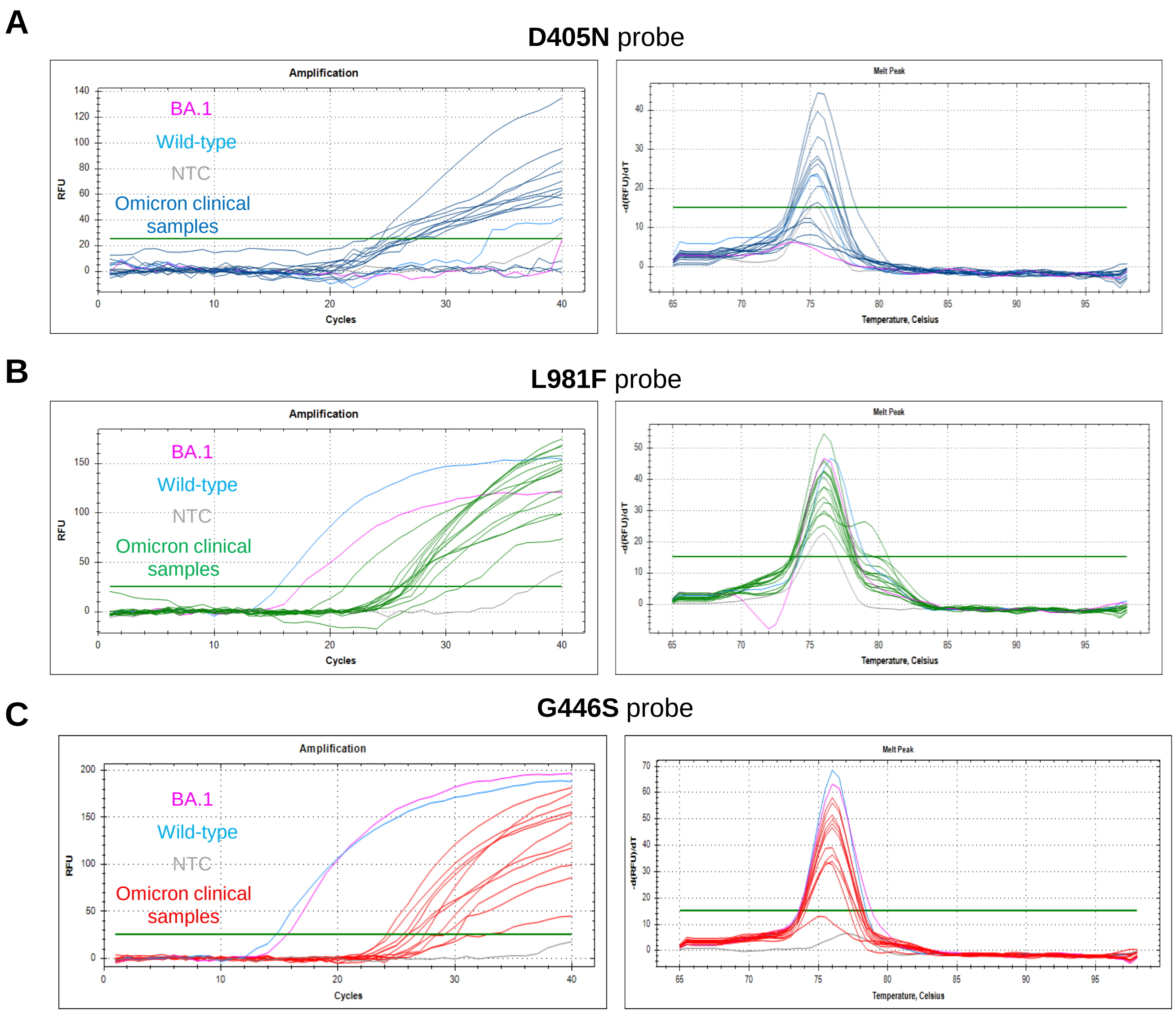

Supplementary Figure 5. Performance and specificity check of D405N, G446S and L981F flanking primers tested on clinical samples. (A), (B) and (C) represent amplification plots and melt curve analyses for D405N, G446S and L981F primer pairs used to prepare single amplicons for Sanger sequencing, respectively (SYBR green chemistry was used.). For all three primers pairs, positive and negative controls included genomic material from Wild-type or Omicron (BA.1) and no template, respectively.

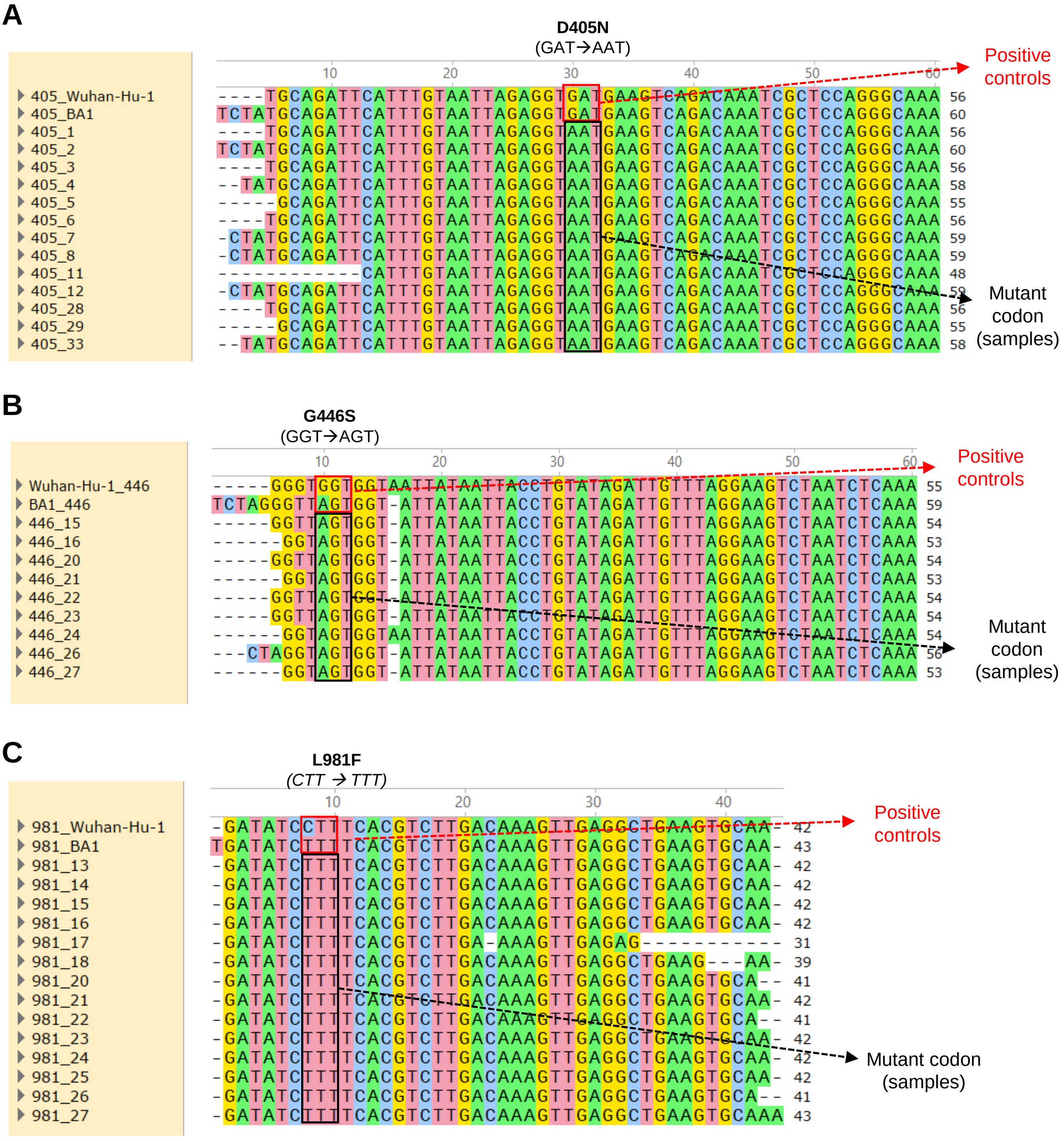

Supplementary Figure 6. Sequence alignment for SNP calling validation for D405N, G446S and L981F probes using Sanger sequencing. Samples were aligned to the appropriate regions in the *S*-gene of the wild-type or Omicron (BA.1) sequences. (A) D405N (GAT→AAT) validation. (B) G446S validation. (C) L981F validation. The wild-type and mutant codons are shown in the red and black boxes, respectively. All clinical specimens contained the mutant codon. Sample numbering corresponds to those used in Figure 5. Nucleotide alignments were performed using SnapGene (Version 6.1.1) using MUSCLE algorithm.
